# Does the British Heart Foundation PocketCPR training application improve confidence in bystanders performing CPR?

**DOI:** 10.29045/14784726.2018.06.3.1.1

**Published:** 2018-06-01

**Authors:** John Renshaw, Georgette Eaton, Pete Gregory, Tim Kilner

**Affiliations:** Coventry University: Orcid ID: https://orcid.org/0000-0002-5774-5877; Oxford Brookes University: Orcid ID: https://orcid.org/0000-0001-9421-2845; University of Wolverhampton: Orcid ID: https://orcid.org/0000-0001-9845-0920; University of Worcester: Orcid ID: https://orcid.org/0000-0001-7725-4402

**Keywords:** bystander, confidence, CPR, PocketCPR

## Abstract

**Objectives:**

Out-of-hospital cardiac arrest has poor prognosis and patients rarely survive unless they receive immediate cardiopulmonary resuscitation from bystanders. In 2012, the British Heart Foundation launched its PocketCPR training application to simplify bystander cardiopulmonary resuscitation training and overcome barriers to resuscitation. This study investigates whether the British Heart Foundation PocketCPR training application improves the confidence of bystanders who perform cardiopulmonary resuscitation during simulated resuscitation attempts.

**Methods:**

This is a mixed method study using a randomised crossover trial with questionnaire analysis. One hundred and twenty participants were randomised to either perform two minutes of cardiopulmonary resuscitation on a resuscitation manikin using the British Heart Foundation PocketCPR application or perform cardiopulmonary resuscitation without instruction. Participants completed a questionnaire to capture their confidence before completing the opposite arm of the study. Each participant then completed a second questionnaire to allow for comparison of levels of confidence.

**Results:**

Participants in this study were more confident in their overall performance of cardio-pulmonary resuscitation using the British Heart Foundation PocketCPR training application compared to performing cardiopulmonary resuscitation without instruction (mean confidence score (0–100): 50.41 with PocketCPR and 43.92 without (p = 0.026)). They were also more confident that the number of chest compressions in this study was correct (mean: 60.39 with PocketCPR vs. 46.10 without (p < 0.001)), and in the delivery of cardiopulmonary resuscitation without having recent cardiopulmonary resuscitation training (mean: 48.67 with PocketCPR vs. 39.79 without (p < 0.002)).

**Conclusion:**

The British Heart Foundation PocketCPR training application improved the confidence of bystanders performing cardiopulmonary resuscitation during simulated resuscitation attempts.

## Introduction

Out-of-hospital cardiac arrest (OHCA) is a global public health problem (Mehra, 2011). Each year, over 60,000 patients suffer OHCA within the UK, yet fewer than 10% of patients survive to hospital discharge (Resuscitation Council (UK), 2015). It is recognised that many of these patients fail to overcome the complex post-cardiac arrest syndrome caused by the abrupt loss of cardiac function; worsened by a lack of immediate resuscitation (Mongardon et al., 2011; Nolan et al., 2015). Patients are more likely to survive OHCA if they receive early recognition of cardiac arrest, immediate cardiopulmonary resuscitation (CPR), prompt defibrillation and effective post-resuscitation care as part of the chain of survival (Nolan, Soar, & Eikeland, 2006). However, the provision of bystander CPR remains unacceptably low within the UK, and public access defibrillation (PAD) has been reported to occur in fewer than 2% of all cases (Deakin, Shewry, & Gray, 2014).

In order to increase the uptake of bystander CPR and improve survival from OHCA, it is necessary to understand and overcome the barriers to the delivery of bystander resuscitation. Previous studies have identified that bystanders are reluctant to perform mouth-to-mouth ventilation due to the perceived risk of infection, have concerns that CPR would not be performed properly, are uneasy about the possible legal consequences of performing chest compressions and are fearful of causing physical harm (American Heart Association, 2014; Coons & Guy, 2009; Lester, Donnelly, & Assar, 2000). In 2012, the British Heart Foundation (BHF) launched its high-profile chest compression-only CPR campaign to encourage lay people to perform chest compressions in OHCA (British Heart Foundation, 2012). The BHF also introduced its PocketCPR training application to provide real-time feedback during CPR and facilitate the delivery of effective chest compression performance in an attempt to alleviate this concern. Although the BHF training application has previously been demonstrated to improve the total number of chest compressions performed during simulated resuscitation attempts (Eaton, Renshaw, Gregory, & Kilner, 2016; Renshaw, Eaton, Gregory, & Kilner, 2017), it is unclear whether it would help bystanders to feel more confident and therefore be more likely to attempt CPR. However, given the persistent variability in rates of survival from OHCA and the figures that show that an estimated 1.4 billion smartphones were sold in 2016 (IDC Research Inc., 2017), there is an unquestionable opportunity for remote digital technology to influence bystander resuscitation.

The value of bystander CPR and early defibrillation has been emphasised in a recent study from the United States. Girotra et al. (2016) noted a marked variation in rates of survival to discharge ranging from 3.4 to 22.0%, and survival with functional recovery ranging from 0.8 to 21.0%. The study identified that rates of bystander CPR and automated external defibrillator use were positively correlated with both outcomes. Similar variability in the likelihood of survival has been demonstrated within the UK, Europe and the rest of the world (Perkins & Cooke, 2012; Strömsöe et al., 2014). With the low provision of bystander CPR and defibrillation, the improvement in OHCA survival has been modest compared to mortality associated with myocardial infarction, stroke and comparable public health concerns (Oranato, Becker, Weisfeldt, & Wright, 2010).

This study aims to establish whether the BHF PocketCPR application improves public confidence during simulated bystander resuscitation attempts. It will use simulated resuscitation attempts since it is not possible to conduct ethical research into the use of the BHF PocketCPR application during real-life cases of OHCA, without the potential to cause harmful delay in treatment. Importantly, simulation facilitates the study of important proxy measures in a safe environment, and this research approach is considered an important and valid alterative.

### Objectives

The aim of this arm of the mixed method study was to investigate whether the BHF PocketCPR training application improves the confidence of bystanders who perform CPR on a training manikin during a simulated cardiac arrest. We hypothesised that lay people would feel more confident in the delivery of bystander CPR, and therefore may be more willing to attempt it, when under instruction from the BHF PocketCPR training application.

## Methods

### Recruitment

Participants were voluntarily recruited from Coventry University campus using a convenience sampling strategy. Each participant was required to be at least 18 years of age and to have not received CPR training within the past six months, as it is recognised that knowledge and confidence in CPR within this time frame may be enhanced (Creutzfeldt, Hedman, Medin, Stengård, & Felländer-Tsai, 2009; Isbye, Meyhoff, Lippert, & Rasmussen, 2007; Woollard et al., 2004). Although participants were recruited from the university campus, volunteers mainly consisted of members of the public who were in or around the city centre campus. Importantly, none of the participants in this study were students on health profession programmes.

### Sample size

The study recruited 120 participants, which is comparable with previous studies investigating OHCA (Woollard et al., 2011). The initial part of the study was a randomised crossover trial to allow for comparison of CPR performance and consider the training benefit of the BHF PocketCPR application, so a sample size calculation was undertaken for that element of the research (Eaton et al., 2016). A sample size of 108 was required to maintain a power of 0.85 and an alpha of 0.05 in the data analysis. All 120 participants completed the qualitative questions.

### Consent and randomisation

Participants were provided with a detailed participant information sheet and had an opportunity to speak to members of the research team before providing written informed consent to participate in the trial. Each of the participants could withdraw their consent at any time without giving reason. Despite this option being available, none of the participants requested to withdraw their consent or made contact with the research team to withdraw their data from this study. A pre-randomised order was generated using PASW statistical software package (version 17.0.2, SPSS Inc., Chicago, IL, USA). Participants were initially invited to either perform CPR using the BHF PocketCPR application or perform CPR without instruction, depending on the pre-randomised order assigned to their participant number.

### Methodology

Resuscitation was performed on a recording Laedral resuscitation manikin to capture the effectiveness of layperson chest compressions (Resusci Anne Skills Station, Laerdal Medical Limited, Orpington, UK) (Figure 1). A simulated resuscitation attempt was chosen to measure the effectiveness of chest compressions without causing undue harm or delay in a real-life situation. Each participant using the BHF PocketCPR application did so on an iPod Touch 2009 device that gave visual and auditory instruction once the application was commenced. Pocket CPR gives visual feedback using accelerometer technology in the form of a display bar indicating current compression depth with a green colour marking the ideal interval, and verbal feedback prompts (including ‘press harder’, ‘press faster’, ‘press slower’, ‘good depth’). Additionally, an integrated metronome signals the correct compression rate of 100 per minute. No feedback is provided on the delivery of rescue ventilations. Participants received no verbal, visual or metronome feedback when performing CPR without the BHF application.

**Figure:**
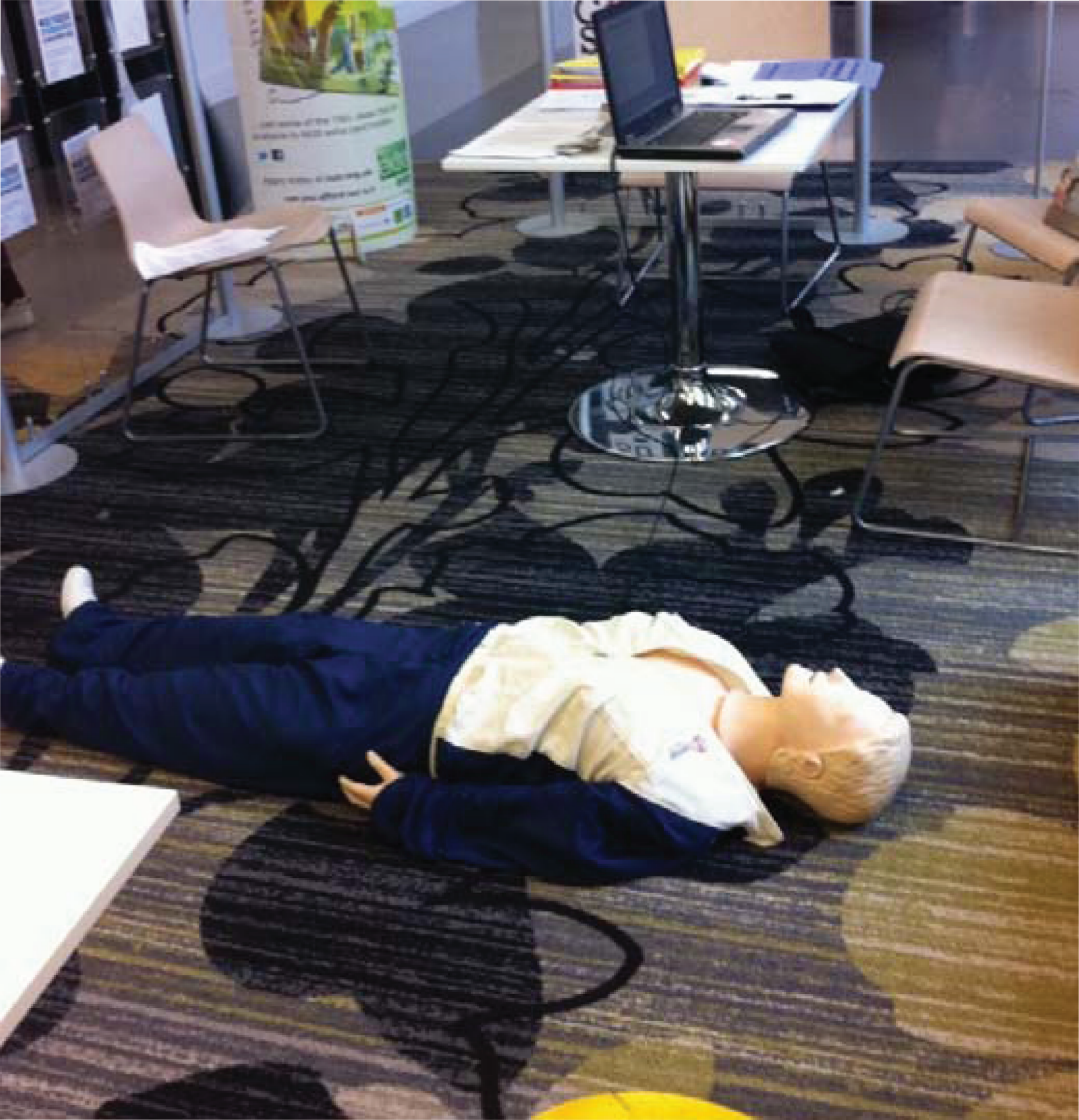
Figure 1. The Laedral resuscitation manikin used to measure the effectiveness of chest compressions in this study.

Between the two arms of the study, participants completed a questionnaire to measure their confidence in performing chest compressions. Confidence was measured using a 100 mm visual analogue scale, and a variety of questions were provided in order to capture information in a range of areas (Supplementary 1). The questions adopted between the two arms of the study were different depending upon which arm the participant followed first. The questionnaire used for participants who had first used the BHF PocketCPR application contained additional questions focusing on the BHF chest compression-only advert, whereas the other subsequent questionnaire did not. Participants were then required to complete the second questionnaire on completion of the second arm of the study to allow for comparison. Demographic data were also gathered to allow for subset analysis (Table 1). Participants rested for two minutes before commencing the second arm of the study to prevent the development of rescuer fatigue.

**Table 1.** Demographic data of participants (n = 120).

**Gender:**	**Total:**
Female	62
Male	58
**Age group (years):**	**Total:**
18–25	69
26–33	11
34–41	8
42–48	22
49+	10

The questionnaire responses were measured and uploaded into IBM SPSS Statistics Version 24 software package to calculate descriptive statistics. Mean values were calculated within the SPSS software, and non-parametric Wilcoxon’s rank tests allowed for the comparison of two related samples. Probability was calculated with a p-value of < 0.05 being considered to be statistically significant.

## Results

All 120 participants completed questionnaires in this study. The baseline demographic profile of participants is presented in Table 1. The table shows almost equal distribution of gender among participants, whereas there was a greater representation of volunteers aged 18–25 than any other age group within our sample.

Participants in this study were more confident in performing CPR without having recent CPR training using the BHF PocketCPR application compared to bystanders who performed CPR without instruction (mean confidence score (0–100): 48.67 with BHF PocketCPR vs. 39.79 without (p < 0.002)) (Renshaw et al., 2017). Although there was a significant difference between those using the application and those who were not, bystander confidence was low in both (using a not confident to very confident scale). In addition, the BHF PocketCPR application also improved confidence in the total number of chest compressions performed by rescuers (mean: 60.39 with BHF PocketCPR vs. 46.10 without (p < 0.001)). However, there was no significant difference between using the BHF PocketCPR application or not in confidence in performing the correct depth of chest compression (mean: 54.57 with BHF PocketCPR vs. 47.38 without (p = 0.21)).

Participants in this study were more confident in their overall performance of bystander CPR using the BHF PocketCPR application during their resuscitation attempts (mean: 50.41 with BHF PocketCPR vs. 43.92 without (p = 0.026)). There was no significant difference in the fear of causing harm to patients in this study (mean: 39.94 with BHF PocketCPR vs. 39.12 without (p = 0.466)). Finally, there was a significant difference in the number of people who felt that their performance of CPR benefitted from the BHF advert for chest compression-only CPR (mean: 48.64 with BHF PocketCPR vs. 39.52 without (p < 0.001), although it is unclear how many participants have seen this advert.

A comparison of these results can be seen in Table 2.

**Table 2.** Results.

Question	Mean		P-value
	Without application	With application	
How confident were you performing CPR without having previous experience?	39.79	48.67	p = 0.002
How confident do you feel that the British Heart Foundation advert for ‘chest compression-only CPR’ improved your performance of CPR in this study?	39.52	48.64	p < 0.001
How confident do you feel about the number of compressions performed in this study being correct?	46.10	60.39	p < 0.001
How confident do you feel about your overall CPR performance?	43.92	50.41	p = 0.026
How confident do you feel about performing CPR and not causing harm to the patient?	39.12	39.94	p = 0.466
How confident do you feel about the depth of compressions performed in this study?	47.38	54.57	p = 0.21

## Discussion

The BHF PocketCPR training application improved bystander confidence in the delivery of CPR without recent CPR training. It appears that participants felt more confident performing CPR using the BHF training application than when they performed CPR without instruction. Previous studies have identified the unquestionable benefits of performing effective chest compressions in cases of OHCA (Hasselqvist-Ax et al., 2015; Holmberg, Holmberg, Herlitz, & Swedish Cardiac Arrest Registry, 2001); therefore, any intervention that may improve the readiness of bystanders to perform chest compressions in cases of OHCA should be considered as an important public health opportunity. Rescuers also felt more confident in the total number of chest compressions performed in this study. The authors have previously established a significant improvement in the total number of chest compressions performed during this simulated resuscitation attempt that reflects the improved confidence in this area (Eaton et al., 2016). Improving the number of chest compressions that are performed is important in maintaining blood flow to vital organs (Georgiou, Papathanassoglou, & Xanthos, 2014) and has been emphasised in the 2015 Resuscitation Guidelines (Soar et al., 2015). Although bystander confidence was higher with the BHF PocketCPR application, it remains concerning that overall confidence remained low in both arms of the study. It would seem that a multi-dimensional approach is required to improve confidence and to increase the number of bystanders who are prepared to attempt CPR.

It was evident that participants were more confident in the number of chest compressions they performed, yet their confidence in achieving the correct depth of compression was low in both groups, with no statistically significant difference found. It is interesting to note that, despite the apparent lack of confidence, the number of chest compressions that achieved the recommended depth was significantly greater when using PocketCPR (Eaton et al., 2016). The explanation for this finding is unclear as the application provides instant feedback to the person performing CPR, so it should raise confidence levels. The literature shows that even trained healthcare professionals perform compressions that are too shallow (Stiell et al., 2012), so it could be that confidence is affected because participants felt that the device was asking them to compress too deeply and increased their risk of doing harm. Current guidelines suggest that a compression depth of 50–60 mm is optimal to achieve the best outcome (Perkins et al., 2015), and this depth is perhaps deeper than many bystanders feel comfortable with. This is a concern, as many people do not attempt CPR for fear of causing injury (Nielsen, Isbye, Lippert, & Rasmussen, 2013), so even though the depth of compression is improved with the application, bystanders may still not commence CPR if they do not feel confident. We have provided our data to the BHF so that they can add it to their website in the hope that it will help to bolster bystander confidence.

Bystanders in this study used chest compression only CPR as per BHF PocketCPR training application instruction in an attempt to overcome a notable barrier to bystander CPR: mouth-to-mouth ventilations (Baldi, Bertaia, & Savastano, 2014; Bobrow et al., 2010; Cabrini et al., 2014). We previously discovered that the removal of rescue ventilations within the BHF PocketCPR arm of the study reduced interruptions in chest compressions performance and improved the consistency of chest compressions (Eaton et al., 2016), with fewer periods of inactivity associated with mouth-to-mouth ventilations. These findings support the current resuscitation guidelines that emphasise the importance of chest compressions that are performed at the correct rate (100–120 compressions per minute) and depth (50–60 mm). These recommendations also stress the importance of limiting episodes where no CPR is performed as these are associated with a poorer chance of survival (Deakin & Koster, 2016; Odegaard, Pillgram, Berg, Olasveengen, & Kramer-Johansen, 2008). Although the BHF PocketCPR application did improve the quality of CPR performed, there was an average initial delay to treatment of 37.31 seconds while participants navigated the application (Eaton et al., 2016), which may be an additional barrier to bystander CPR through lack of digital fluency. It also presents a limitation in the design of the application that requires further consideration. Most smartphones allow an application to be permanently available on the home screen, but this is generally an option selected by the owner of the device. It may be possible to develop the application in such a way that it is installed on the home page during the download; that would reduce the time spent scrolling to find the application at a time-critical moment.

Fear of causing harm and potential litigation are well-established barriers to bystander CPR (Sasson et al., 2013; Schmid et al., 2016). Our results contribute to this body of evidence and indicate that participants were not confident that harm would not be caused in performing chest compressions with or without PocketCPR. While these results do not meet significance, fear of causing harm displayed the lowest confidence reported in our study and this identifies a significant problem in attitudes towards performing CPR. The authors note that a nationwide sustainable public health campaign may alleviate concerns surrounding these fears. However, previous UK campaigns have existed, yet their effect on bystander confidence in cardiac arrest remains unmeasured (British Heart Foundation, 2014; Resuscitation Council (UK), 2014).

An important factor to consider in any simulated resuscitation attempt is the progression of levels of confidence of bystanders performing CPR during simulation, into the willingness of bystanders performing CPR in real-life situations. The authors recognise that while the bystanders may feel more confident using the BHF PocketCPR to perform chest compressions on a resuscitation manikin, they may not be willing to perform CPR in real-life cases of OHCA. Furthermore, additional barriers such as an emotional attachment to the victim, a reluctance to perform CPR in the presence of excessive vomit or a fear of breaking ribs may, specifically, limit the willingness of bystanders to perform CPR despite the use of the BHF PocketCPR. It is not possible to suggest that the BHF PocketCPR application can overcome these additional barriers and improve the willingness of bystanders to perform CPR. Consequently, further investigation is required.

Lastly, as this study is a simulated resuscitation attempt using a recording resuscitation manikin, the levels of confidence reported cannot be directly transferred into patient outcomes and chances of survival. Real-life bystander CPR is known to be more stressful and emotive, which will influence the likelihood and willingness of bystanders to perform CPR. Nevertheless, these proxy measures remain important and valid results that were obtained using a recognised research method.

## Limitations

This study had a number of limitations. Firstly, it requires participants to be able to operate a smartphone in order to commence the PocketCPR application and the authors recognised this is not a universal skill. Moreover, it is recognised that the device requires the pre-download of the BHF PocketCPR application, which could reduce the opportunity to use this tool.

Secondly, the sampling methodology attracted a larger proportion of younger participants between the ages of 18 and 25 which may impact upon their ability to perform adequate chest compressions and their confidence in the use of smartphone technology. In addition, the use of a crossover manikin study creates an opportunity for participants who performed CPR using the BHF PocketCPR application to gain some form of training benefit from performing CPR compared to those who perform CPR without instruction. However, as this device is a training application then this may be useful information to derive from in further analysis.

Finally, the results of this study are considered as proxy measures that cannot be linked to patient outcomes. Therefore, the generalisability of the study is impacted upon by its simulated manikin study design.

## Conclusion

In conclusion, the BHF PocketCPR training application improved the levels of confidence of bystanders who performed CPR during a simulated resuscitation attempt following at least six months without CPR training. In addition, bystanders were significantly more confident that they performed the correct number of chest compressions and with their overall CPR performance using the PocketCPR application. Although bystanders were not more confident in the depth of chest compressions performed, the BHF PocketCPR application did improve both the number of chest compressions performed and number of compressions to achieve an adequate depth of 50–60 mm in this mixed method study.

The results of this study provide a novel evaluation of the BHF PocketCPR training application in bystander CPR training. Paramedics are dependent upon the delivery of prompt and effective bystander CPR prior to their arrival, so an evaluation of a tool that is designed to improve the performance of bystander CPR is useful. Ultimately, any attempt to improve the level of confidence of bystanders who undertake CPR, either in training or in real life, may help encourage the provision of bystander CPR during real-life OHCA. That being said, further research and education is required to reduce the fear of causing harm to patients suffering OHCA and to explore the opportunity in the use of smartphone technology in pre-hospital resuscitation.

## Acknowledgements

The authors would like to thank Nicola Reeve (NR) from Coventry University for her assistance in the statistical analysis used within this study.

## Author contributions

All of the named authors (JR, GE, PG, TK) were involved in the planning, acquisition of data and construction of the research methodology. JR and GE were involved in the main drafting of this manuscript. JR performed statistical analysis under the supervision of NR. PG and TK performed substantial redrafting of this manuscript prior to submission. Each of the named authors fulfil the full criteria for authorship.

## Conflict of interest

None declared.

## Ethics

This trial received ethical approval from the Coventry University Ethics (P4090). Participants provided their informed written consent to participate in this study.

## Funding

None.
